# Analysis of the microRNA expression profiles of chicken dendritic cells in response to H9N2 avian influenza virus infection

**DOI:** 10.1186/s13567-020-00856-z

**Published:** 2020-10-17

**Authors:** Jing Yang, Xinmei Huang, Yuzhuo Liu, Dongmin Zhao, Kaikai Han, Lijiao Zhang, Yin Li, Qingtao Liu

**Affiliations:** 1 Key Laboratory of Veterinary Biological Engineering and Technology of Ministry of Agriculture, National Center for Engineering Research of Veterinary Bio-products, Institute of Veterinary Medicine, Jiangsu Academy of Agricultural Sciences, 50 Zhongling Street, Nanjing, 210014 Jiangsu China; 2 Jiangsu University, Zhenjiang, China

**Keywords:** H9N2 avian influenza virus, microRNA, chicken dendritic cell, pathogenesis, host defense response

## Abstract

MicroRNA (miRNA) plays a key role in virus-host interactions. Here, we employed deep sequencing technology to determine cellular miRNA expression profiles in chicken dendritic cells infected with H9N2 avian influenza virus (AIV). A total of 66 known and 36 novel miRNAs were differently expressed upon H9N2 infection, including 72 up-regulated and 30 down-regulated miRNAs. Functional analysis showed that the predicted targets of these miRNAs were significantly enriched in several pathways including endocytosis, notch, lysosome, p53, RIG-I-like and NOD-like receptor signaling pathways. These data provide valuable information for further investigating the roles of miRNA in AIV pathogenesis and host defense response.

## Introduction, methods and results

H9N2 AIV has been circulating worldwide in multiple avian species and is endemic in poultry populations across Eurasia. On poultry farms, H9N2 AIV could result in a decrease in growth performance and egg production, and reduce the efficacy of vaccine interventions, and cause serious disease and even death with secondary infections of bacterial or viral pathogens [1, 2]. Although great efforts have been made to develop intervention strategies to control H9N2 AIV infections in poultry, including a vaccination program with inactivated vaccines in China, H9N2 AIV outbreaks have continued to occur over the past two decades. Significantly, poultries have served as key intermediates in the transmission of AIV from avian species to humans, and H9N2 AIV has occasionally been transmitted from poultries to humans [1]. More seriously, H9N2 AIV has donated their internal genes to other subtype AIVs and facilitated the genesis of other emerging human-lethal AIVs, such as H5N1, H7N9, H10N8 and H5N6 AIVs [1]. Therefore, understanding the interaction mechanism between H9N2 AIVs and chickens is not only essential to the control of avian influenza in poultry, but also important for human health.

MiRNAs are non-coding RNAs with a length of about 22 nucleotides, and could regulate gene expression by base pairing with the 3′- or 5′-UTR of the target mRNAs. They have been shown to be implicated in several cellular functions, including proliferation, differentiation, tumorigenesis, apoptosis, immune and inflammatory response, etc. An increasing number of studies showed that influenza virus infection can trigger changes of cellular miRNA profiles. For example, differential miRNA profiles were found in mouse lungs infected with the 1918 pandemic H1N1 and seasonal H1N1 influenza viruses [3]. Another study revealed strain-specific host miRNA molecular signatures associated with the swine-origin H1N1 and avian-origin H7N7 influenza A virus in human A549 cells [4]. Differential miRNA expression profiles have been observed in chicken lungs during H5N3 AIV infection [5]. In addition, H9N2 AIV infection has been shown to activate the immune responses of mouse and avian dendritic cells by regulating the expression of miRNAs [6–8]. All of these studies suggest that miRNAs play an important role in the complex interactions occurring between influenza viruses and their hosts [9]. However, such studies of H9N2 AIV have focused on a very limited number of miRNAs using reverse transcription quantitative PCR (RT-qPCR) or traditional microarray analyses.

Dendritic cells (DCs) are able to sense invading viruses and play a key role in the host defense response to virus infection [10]. Upon encountering viral pathogens, DCs produce interferons (IFN) and other regulatory cytokines that contribute to the innate immune response, and then migrate to secondary lymphoid organs and present antigens to T cells to induce adaptive immune responses [10]. However, some viral pathogens can induce the dysregulation of DC function, which in turn influences immunological homeostasis and the clinical outcome of infection [11]. Since H9N2 AIV could influence the host response to vaccine and the outcome of secondary infections, it is necessary to study the interaction between DCs and H9N2 AIV. In this study, we determined the global miRNA expression profiles in chicken DCs during H9N2 AIV infection using deep sequencing technology for the first time, which may provide helpful insights into understanding the interaction between DCs and H9N2 AIV.

Chicken DCs were cultured from bone marrow cells with RPMI-1640 medium containing 5% FBS (Wisent Bio Products, Canada), 50 ng/mL chicken GM-CSF (Abcam, USA) and 10 ng/mL IL-4 (Kingfisher, USA), as previously described [12]. To identify miRNA changes of DCs infected with H9N2 AIVs, two small RNA libraries were constructed in triplicates for H9N2 AIV-infected (A/duck/Nanjing/06/2003 strain, with a multiplicity of infection of 5) and mock-infected DCs at 6 h post infection, and then were sequenced by Solexa technology on Illumina HiSeq XTen (Illumina, USA). Sequencing data have been submitted to the GEO database (accession number GSE147658). After removing low quality sequences, adapter sequences, and sequences smaller than 18 nt, 6.76–10.49 and 10.38–10.67 million clean reads were obtained from the virus and mock infected groups. The length distribution of the clean reads was similar in infection and mock libraries, and the majorities ranged from 22 nt to 23 nt in size (Additional file 1), which indicates the successful enrichment of mature miRNAs in the libraries of the two groups. These clean reads were then screened against the GenBank database and Rfam database, and more than 80% of the annotated small RNAs (miRNA, tRNA, rRNA, and other non-coding RNA) were categorised as miRNAs (Additional file 2). Next, these clean reads were aligned with chicken miRNAs in miRBase version 21.0 database, and 4.29–8.12 and 6.72–7.37 million clean reads were mapped to the known miRNA of chicken in H9N2 AIV and mock infected libraries respectively (Additional file 3). The rest reads, which were mapped to chicken genome, were further utilized to predict novel miRNAs using miRDeep2 software as described previously [13]. Finally, we identified 994 known and 9208 novel miRNAs in all libraries (Additional file 4).

To identify the differentially expressed miRNAs between H9N2 AIV-infected and mock-infected groups, the raw counts of miRNA reads were further normalized by transcripts per million reads (TPM), and the miRNA expression levels between the two groups were compared using the DESeq R package [14]. The estimated absolute log2-fold change of > 1, and a corrected p-value < 0.05 were used as the thresholds for significant differently expressed genes. The results showed that 66 known and 36 novel miRNAs were significantly differentially expressed between the two groups (Table 1, Additional file 5). Of these miRNAs, 42 known and 30 novel miRNAs were up-regulated, and 24 known and 6 novel miRNAs genes were down-regulated following H9N2 AIV infection.

**Table 1:** Significantly differentially expressed known chicken miRNA in DCs induced by H9N2 AIV infection

miRNA	H9N2-infected DCs (TPM)	Mock-infected DCs (TPM)	Fold change	*P* value	Style
gga-miR-302b-3p	8.800102678	0.321066631	27.41	1.03E−02	Up
gga-miR-302d	7.394757730	0.420683683	17.58	2.80E−02	Up
gga-miR-551-5p	16.17959332	0.963199892	16.79	2.76E−04	Up
gga-miR-449c-5p	30.39688718	2.132361815	14.26	7.23E−07	Up
gga-miR-551-3p	326.4538591	44.31762933	7.37	7.29E−31	Up
gga-miR-1666	15.19538833	2.457331401	6.18	7.86E−03	Up
gga-miR-187-3p	166.0448253	30.56495477	5.43	1.19E−15	Up
gga-miR-205b	13.92136269	2.579163928	5.39	2.85E−02	Up
gga-miR-1467-3p	223.8620766	49.12096621	4.56	6.74E−08	Up
gga-miR-365-3p	1805.885624	405.3167326	4.46	1.19E−32	Up
gga-miR-365b-5p	894.8004725	206.3600374	4.34	2.42E−14	Up
gga-miR-1708	14.70678182	3.420531293	4.29	4.00E−02	Up
gga-miR-21-3p	2753.261063	713.6833743	3.86	1.28E−30	Up
gga-miR-190a-3p	167.0188522	47.11513315	3.55	2.81E−08	Up
gga-miR-190a-5p	630.0087664	197.5629264	3.19	9.54E−17	Up
gga-miR-7467-3p	85.99901053	27.24794349	3.16	9.94E−06	Up
gga-miR-200a-3p	238.1628792	75.72797869	3.14	1.80E−11	Up
gga-miR-193a-3p	217.6650688	71.21556278	3.06	1.03E−05	Up
gga-miR-3535	274.4292255	91.00293488	3.02	1.33E−11	Up
gga-miR-29b-3p	86081.05620	29667.27626	2.90	2.39E−21	Up
gga-miR-103-1-5p	72.87844804	25.41928950	2.87	2.80E−02	Up
gga-miR-7467-5p	92.88247498	32.45298254	2.87	5.38E−06	Up
gga-miR-22-5p	10483.71708	3915.882453	2.68	2.26E−17	Up
gga-miR-451	5731.056244	2160.176030	2.65	1.05E−04	Up
gga-miR-20a-5p	40364.26188	15575.49639	2.59	8.40E−12	Up
gga-miR-155	26783.80295	10737.39804	2.49	1.19E−15	Up
gga-miR-1434	685.0883524	276.4517753	2.48	5.54E−09	Up
gga-miR-7	1759.477832	751.2428887	2.34	2.98E−11	Up
gga-miR-429-3p	326.3798282	139.7225717	2.34	3.99E−08	Up
gga-miR-200b-3p	125.0793544	53.86359132	2.32	5.96E−05	Up
gga-miR-29a-5p	571.6283718	250.7912729	2.28	3.55E−09	Up
gga-miR-15b-5p	13909.71515	6196.684286	2.24	1.77E−12	Up
gga-miR-33-5p	4032.377244	1814.920557	2.22	1.59E−10	Up
gga-miR-215-5p	266.1401100	121.3765857	2.19	6.69E−07	Up
gga-miR-101-2-5p	632.9850972	292.6375455	2.16	3.96E−08	Up
gga-miR-32-5p	2061.528955	955.6190685	2.16	1.35E−10	Up
gga-miR-153-3p	206.7604279	95.94182016	2.16	7.22E−04	Up
gga-miR-181b-5p	10563.99373	4909.109999	2.15	3.30E−06	Up
gga-miR-30e-5p	39176.41147	18594.68260	2.11	4.17E−08	Up
gga-miR-15a	24351.55946	11588.38769	2.11	8.35E−11	Up
gga-miR-22-3p	37910.33000	18564.17097	2.04	1.59E−10	Up
gga-miR-181a-5p	34649.54269	17283.22335	2.00	5.70E−08	Up
gga-miR-6594-5p	94.67578132	197.5792320	− 2.09	5.89E−05	Down
gga-miR-6642-5p	23.43620583	52.39678050	− 2.24	2.11E−02	Down
gga-miR-19b-5p	464.0802376	1045.118019	− 2.25	2.03E−11	Down
gga-miR-6556-3p	43.50229656	101.8832443	− 2.34	2.50E−04	Down
gga-miR-1712-5p	24.56364507	57.59468264	− 2.34	1.11E−02	Down
gga-miR-6696-3p	55.97845749	136.4756894	− 2.44	1.04E−02	Down
gga-miR-1559-3p	571.3932734	1401.389558	− 2.45	2.33E−13	Down
gga-miR-15b-3p	53.47288137	134.9119737	− 2.52	5.41E−06	Down
gga-miR-1759-3p	15.33179667	41.36731788	− 2.70	1.02E−02	Down
gga-miR-130b-3p	7672.726337	21287.07056	− 2.77	9.60E−18	Down
gga-let-7a-2-3p	19.28809129	53.87717145	− 2.79	1.44E−03	Down
gga-miR-34c-5p	7.702176123	23.13412006	− 3.00	4.81E−02	Down
gga-miR-365-2-5p	15.08675579	53.42821336	− 3.54	3.28E−04	Down
gga-miR-15c-3p	5.398144233	20.51538185	− 3.80	2.33E−02	Down
gga-miR-92-5p	82.93267399	316.4139231	− 3.82	2.53E−14	Down
gga-miR-193b-5p	5.710651656	22.56361416	− 3.95	1.15E−02	Down
gga-miR-184-3p	4.083834071	16.96911507	− 4.16	4.11E−02	Down
gga-let-7b	141.4738821	623.2702767	− 4.41	3.18E−26	Down
gga-miR-128-1-5p	26.18173380	119.0613949	− 4.55	6.81E−11	Down
gga-miR-31-3p	6.390196622	33.92087134	− 5.31	1.95E−04	Down
gga-miR-6575-3p	3.409378134	20.08863924	− 5.89	2.31E−02	Down
gga-miR-1591-3p	1.921727259	11.45189329	− 5.96	3.79E−02	Down
gga-miR-31-5p	70.91080554	448.1771463	− 6.32	1.54E−12	Down
gga-miR-1702	19.45910134	203.4676260	− 10.46	1.20E−07	Down

**Figure 1:**
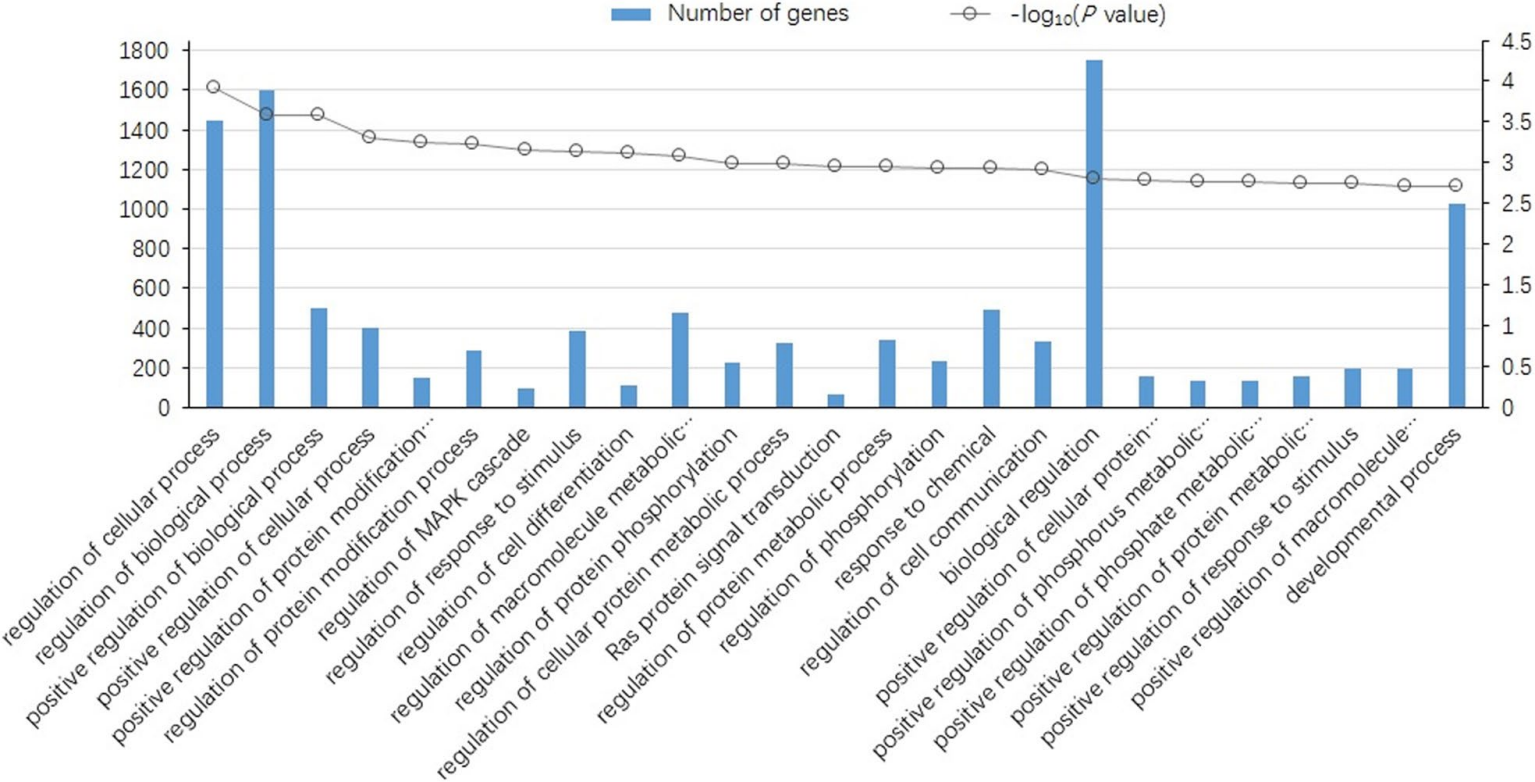
**Gene Ontology (GO) of the top 25 biological processes enriched by predicted target genes from SDE miRNAs.** The horizontal axes denote the GO terms. The vertical axes represent the number of DEGs (left) and the −log (P value) (right) for each term. A full list of GO terms is shown in Additional file 6.

**Figure 2:**
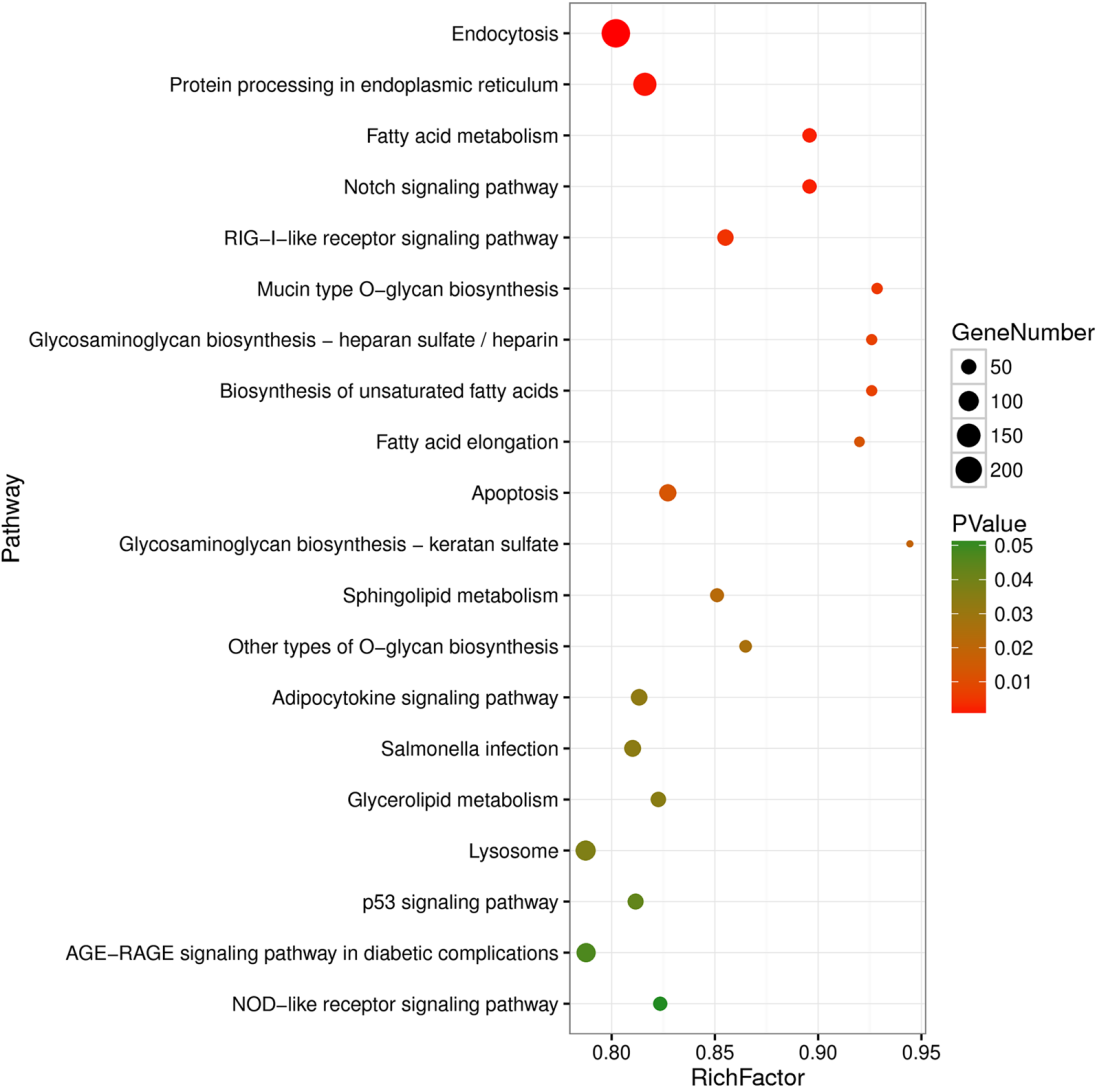
**Top 20 KEGG pathways enriched by predicted target genes from SDE miRNAs.** The color scale and the circle on the right–hand side illustrate the significant level and target gene number of the indicated pathway. A full list of pathways is shown in Additional file 7.

To understand the biological function of miRNAs during H9N2 AIV infection, 13503 target genes were predicted for the significantly differentially expressed (SDE) miRNAs using RNAhybrid and miRanda software [15], and one miRNA targeted many mRNA and vice versa (Additional file 6). GO enrichment analysis of the target genes showed that the SDE miRNAs were involved in the regulation of cellular process, protein modification process, MAPK cascade, response to stimulus, protein metabolic process, and other processes (Figure 1, Additional file 7). To analyze the roles of these SDE miRNA in regulatory networks, KEGG pathway analysis was also performed for the target genes. The results showed that these targets were mainly involved in endocytosis, notch signaling pathway, RIG-I-like receptor signaling pathway, lysosome, p53 signaling pathway, and NOD-like receptor signaling pathway (Figure 2, Additional file 8). These results indicate that the SDE miRNA may play a crucial role in regulating the cellular metabolic process, signal transduction and immune responses of DCs during H9N2 AIV infection.

**Figure 3:**
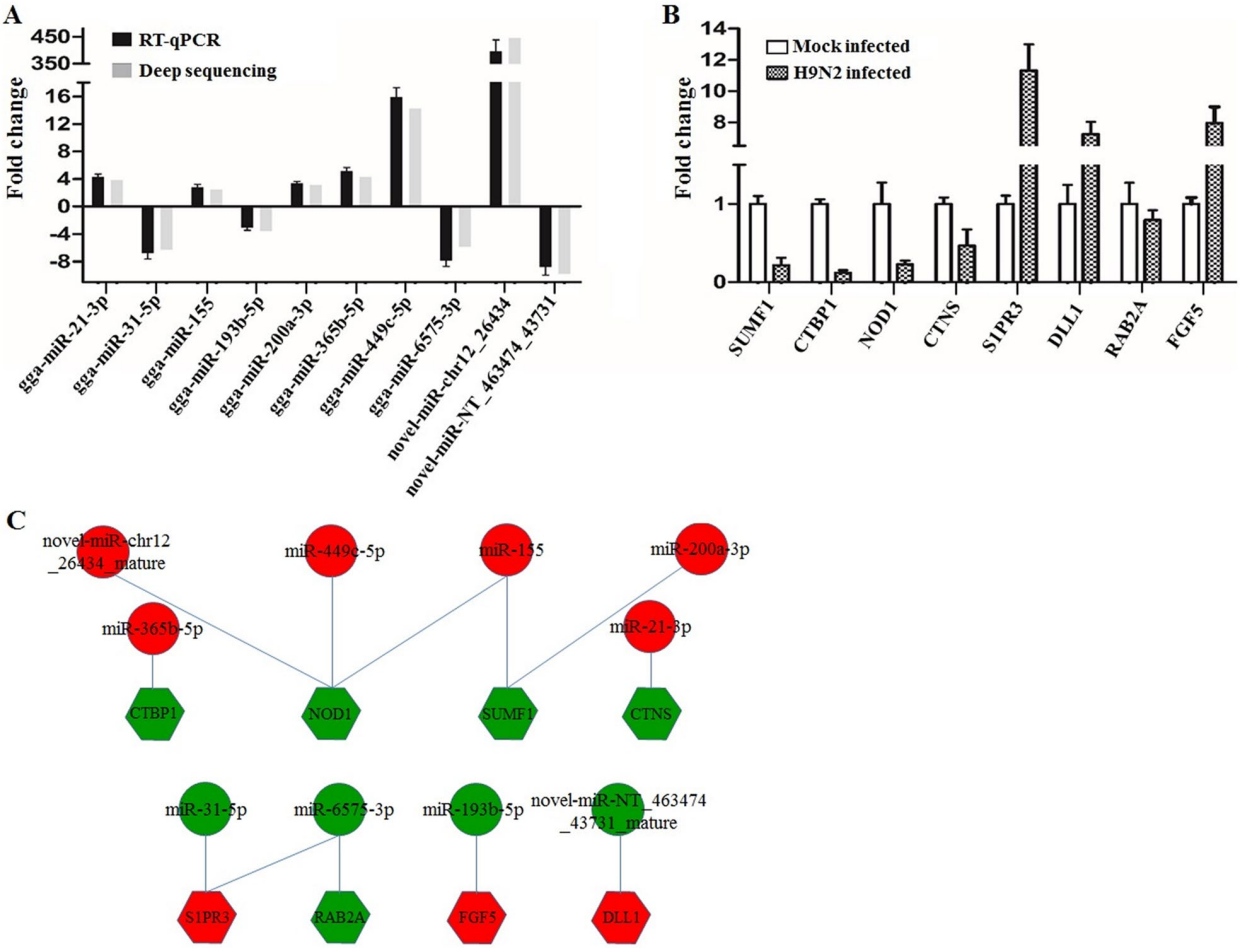
**Validation of miRNAs and their mRNA targets expression by quantitative RT-PCR.** The expression changes of 10 miRNAs (**A**) and their mRNA targets (**B**) in the H9N2 AIV-infected DCs was calculated using the 2^−ΔΔCT^ method and represented as the n-fold change relative to the mock-infected DCs. The 5 s and β-actin genes were used as the reference genes for miRNA and mRNA respectively. **C** Relationships between miRNAs and mRNA targets. Red indicates the up-regulated miRNAs (circle) and target mRNAs (hexagon); green indicates the down-regulated miRNAs and target mRNAs.

To confirm the data obtained through RNA-seq analysis, the miRNAs were isolated from a replica RNA sequencing infection experiment using miRNA isolation kit (TIANGEN, China). The isolated miRNAs were first polyadenylated with polyA polymerase and then were reverse transcribed into complementary DNA (cDNA) with a poly(T) adapter primer (TIANGEN, China). Ten SDE miRNAs were selected for validation by quantitative reverse transcription PCR (qRT-PCR) using the miRNA qPCR SYBR Green Detection Kit (TIANGEN, China). The miRNA-specific forward primers used in this study are shown in Additional file 9. The results confirmed the up-regulation of five known and one novel miRNAs, and the down-regulation of three known and one novel miRNAs in H9N2 AIV-infected DCs compared with the mock cells (Figure 3A). The results of qRT-PCR were consistent with the data obtained from RNA-seq, although larger fold change values were obtained from qPCR for some selected miRNAs.

Finally, eight target mRNAs for the 10 SDE miRNAs were also selected for qRT-PCR analysis (Figure 3B). The results showed that SUMF1, CTBP1, NOD1, and CTNS were down-regulated, and S1PR3, DLL1, and FGF5 were up-regulated, which was inversely correlated with the expression of their miRNAs (Figure 3C). However, the expression of other one gene (RAB2A) was positively correlated with the expression of their miRNAs.

## Discussion

In recent years, high-throughput sequencing technology has been effectively used to identify differentially expressed miRNAs, on a genome-wide scale, during viral infection. Increasing studies showed that miRNAs, as ubiquitous regulators of gene expression, play an important regulatory role in virus-host interactions. Nevertheless, the roles of miRNA in the regulation of host responses to H9N2 AIV infection in chicken DCs are poorly understood. In the present study, high-throughput sequencing approach was subjected to identify differentially expressed miRNAs in chicken DCs in response to H9N2 AIV infection. A total of 66 known and 36 novel differentially expressed miRNAs were identified successfully. Among these 66 know SDE miRNAs, 12 miRNAs (miR-22-3p, miR-22-5p, miR-30e-5p, miR-31-5p, miR-32-5p, miR-33-5p, miR-92-5p, miR-155, miR-184-3p, miR-215-5p, miR-451 and let-7b) are also found to be differentially expressed in chicken lungs, immune organs, and embryo fibroblasts during H5N3, H5N1 and H9N2 AIV infection [5, 16, 17]. In addition, another 7 know SDE miRNAs (miR-7, miR-21-3p, miR-34c-5p, miR-187-3p, miR-200a-3p, miR-429-3p and miR-1434) are identified in other virus infected chickens [18–20]. Therefore, these DE miRNAs might play a vital role in the interaction between chicken DCs and H9N2 AIV.

The innate immune responses are the first line of host defense against virus infection. Emerging data have showed some miRNAs can inhibit or promote virus replication by regulating host innate immune responses. Several of the SDE miRNAs identified in this study can target genes that are associated with immune responses. It has been reported that miR-7 is widely conserved in animal species and is up-regulated during invertebrates and vertebrate’s virus infection, such as poliovirus and white spot syndrome virus (WSSV). In crab, the miR-7 could inhibit host anti-viral immune response by targeting Myd88 to enhance WSSV replication [21], whereas the miR-7 up-regulation induced the inhibition of poliovirus infection in human cells [22]. Similarly, miR-7 was also found to be up-regulated in human influenza virus infection [23] and in H9N2 AIV infection in this study. Additionally, miR-21-3p has been found to be down-regulated during H5N1 AIV and 2009 pandemic H1N1 influenza virus infection and could promote influenza virus replication by repressing the expression of HDAC8 gene in A549 cells [24]. But, the miR-21-3p was found to be up-regulated in chicken DCs during H9N2 virus infection in the present study. Therefore, the role of miR-7 and miR-21-3p in H9N2 virus infection needs to be further studied.

In addition, two DE miRNAs, miR-155 and miR-130b-3p, identified in this study have been reported to have an antiviral activity in chicken cells. As one of the widely studied miRNAs, miR-155 is generally believed to be a multifunctional miRNA and plays a critical role in cancer, immune and inflammation response, and viral infection. It was reported that miR-155 is required for the function of B and T lymphocytes and dendritic cells [25], and the induction of this microRNA negatively modulates host innate immune responses and suppresses Japanese encephalitis virus replication in human microglial cells [26]. However, miR-155 was found to suppress the TLR3 expression in chicken embryo fibroblast cells and HD11 cells [27], and contributed to the increased susceptibility to Marek’s disease in chickens [28]. However, other studies showed that the miR-155 enhanced type I interferon expression via targeting SOCS1 and TANK, and suppresses infectious bursal disease virus replication in DF1 cells [29]. The miR-130b-3p belongs to the miR-130/301 family and has been found to take part in the regulation of cytokine expression. A recent study showed that the miR-130b-3p could target socs5 to enhance the expression of STAT in chicken DF1 cells, which contributes to the increase of IFN-β and, further suppresses the replication of infectious bursal disease virus [30]. Therefore, it is worth for further study whether these microRNAs play a regulatory role in H9N2 AIV infection via regulating host innate immune response.

Some miRNAs are able to target multiple mRNAs, which may be involved in the regulation of multiple cell processes. GO enrichment analysis of the potential targeted genes showed that these DE miRNAs are mainly involved in the regulation of metabolic process, signal transduction and immune response (Additional file 6). The KEGG pathway analysis showed that DE miRNAs are involved in the regulation of endocytosis, Notch signaling pathway, RIG-I like receptor signaling pathway, lysosome, p53 signaling pathway, and other pathways (Additional file 7). The Notch signaling pathway is known as a well-conserved throughout metazoans, and plays a fundamental role during embryonic development that is associated with cell fate determination, and immune regulation. It is known that various viruses can exploit the Notch signaling pathway to regulate viral replication and affect the fate of infected cells. In human, HIV could inactivate Notch signaling to result in the inhibition of KSHV lytic replication and the induction of pro-proliferative and -survival cytokines, such as IL-2 and TIMP-1 [31]. Conversely, influenza virus infection activated Notch signaling by up-regulating the Notch ligand Delta-like 1 expression in mice macrophages, and blocking of Notch signaling led to higher virus load with an impaired production of IFN-γ in mice lungs [32]. Although the Notch signaling pathway has been reported to be involved in regulation of cell proliferation and differentiation in chickens [33], there are no reports about the effects of this pathway in virus infection in chickens. Therefore, further work is required to determine the specific roles of Notch signaling in the interaction between H9N2 virus and chicken DCs. Lysosomes are acidic and hydrolytic organelles within cells, which are known primarily to degrade macromolecules or infected pathogens delivered by endocytosis, phagocytosis, and autophagy, and play vital roles in innate immunity recognition, antigen presentation, and pathogen elimination [34, 35]. In the current study, 100 genes of the lysosome pathway were predicted to be targeted by 91 DE miRNAs. These results suggested that these miRNAs might be involved in the regulation of the innate immune response and antigen presentation functions of DCs by targeting the lysosome pathway during H9N2 infection.

In summary, the miRNA expression profiles in chicken DCs upon H9N2 AIV infection was evaluated by deep sequencing. A total of 66 known differentially expressed miRNAs and 36 novel miRNA candidates were identified, supporting the point that certain miRNAs are essential in host and virus interaction. Target prediction and functional analysis showed that these miRNAs may be involved in the regulation of host defense response and viral replication during H9N2 AIV infection. However, further research is needed to investigate the specific role of these miRNAs during H9N2 infection in DCs.

## Supplementary information

**Additional file 1. Length distribution of the clean reads in libraries from H9N2-infected and mock-infected DCs.** The majority of the small RNAs in all libraries were at 22–23 nt. M: mock-infected DCs; V: H9N2 AIV-infected DCs.

**Additional file 2. Pie charts of small RNAs percentages in libraries from H9N2-infected and mock-infected DCs.** More than 80% of the annotated small RNAs in all libraries were miRNAs. M: mock-infected DCs; V: H9N2 AIV-infected DCs.

**Additional file 3. Summary of small RNAs sequencing data.** M: mock-infected DCs; V: H9N2 AIV-infected DCs.

**Additional file 4. MiRNA profile of H9N2-infected and mock-infected DCs.** In total, 994 known and 9208 novel miRNAs were identified in all samples.

**Additional file 5. Significantly differentially expressed (SDE) novel chicken miRNA in DCs induced by H9N2 AIV infection.** Thirty-six novel miRNAs were found to be significantly differentially expressed between H9N2-infected and mock-infected DCs

**Additional file 6. Predicted targets for SDE miRNAs.** Using RNAhybrid and miRanda software, 13503 target genes were predicted for SDE miRNAs, and one mRNA was targeted by many miRNAs and vice versa.

**Additional file 7. GO terms for biological process enriched by the predicted target genes from SDE miRNAs.** GO enrichment analysis revealed 180 biological processes were significantly (p-value < 0.05) enriched by target genes, and the 27 terms associated with immune responses were marked in bold.

**Additional file 8. KEGG pathway annotations for the predicted target genes from SDE miRNAs.**

**Additional file 9. Primers used to detect miRNA and target mRNA expression levels with quantitative RT-PCR.**

## Data Availability

Sequences were deposited at NCBI GEO repository with Accession Number GSE147658.

## Abbreviations

AIV: avian influenza
cDNA: complementary DNA
DCs: dendritic cells
IFN: interferons
miRNA: microRNA
qRT-PCR: quantitative reverse transcription PCR
SDE: significantly differentially expressed
TPM: transcripts per million reads
WSSV: white spot syndrome virus
