# Supplementary figures and images for: Analysis of the microRNA expression profiles of chicken dendritic cells in response to H9N2 avian influenza virus infection
Source: Vet Res. 2020 Oct 17;51:132. doi: 10.1186/s13567-020-00856-z (PMC7568386; doi:10.1186/s13567-020-00856-z)

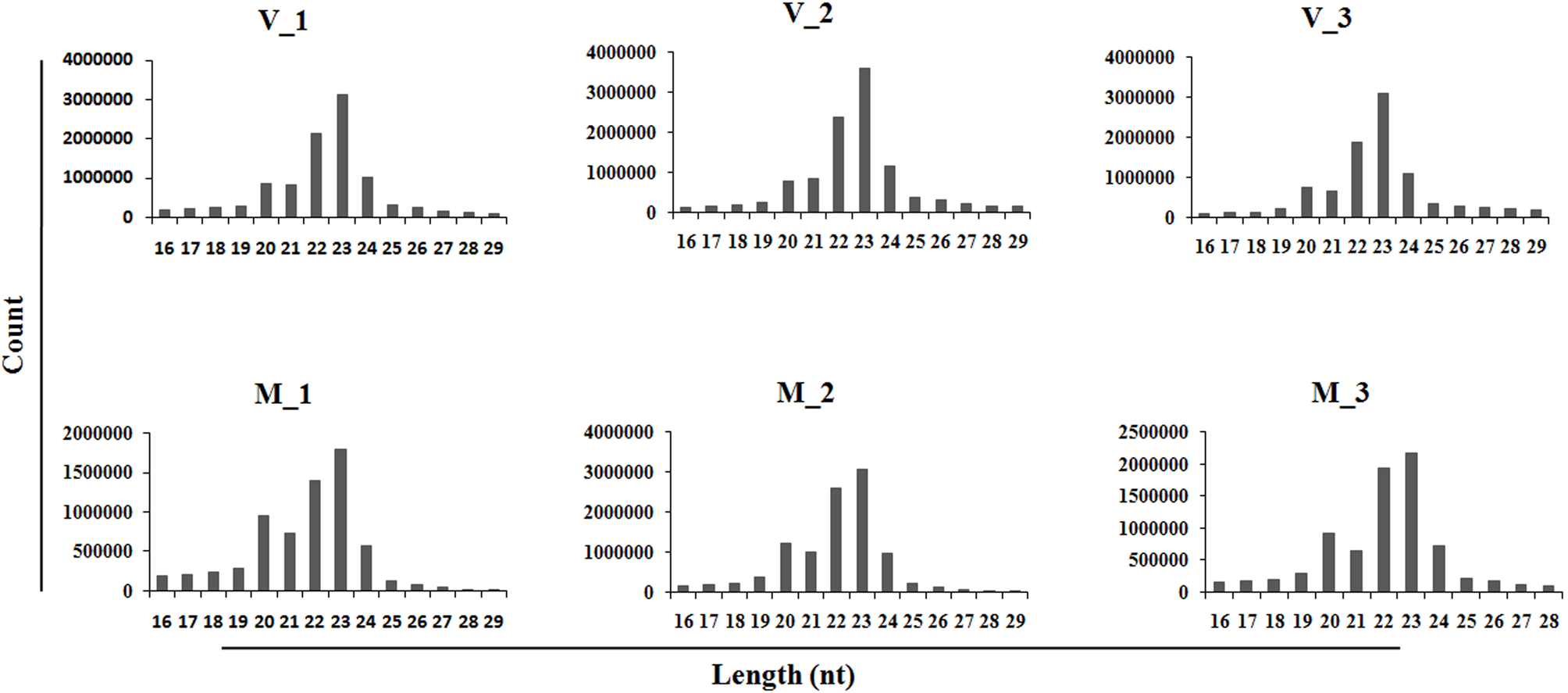

Supplement: Supplementary file 1 — Additional file 1. Length distribution of the clean reads in libraries from H9N2-infected and mock-infected DCs. The majority of the small RNAs in all libraries were at 22–23 nt. M: mock-infected DCs; V: H9N2 AIV-infected DCs. [file 13567_2020_856_MOESM1_ESM.tif]

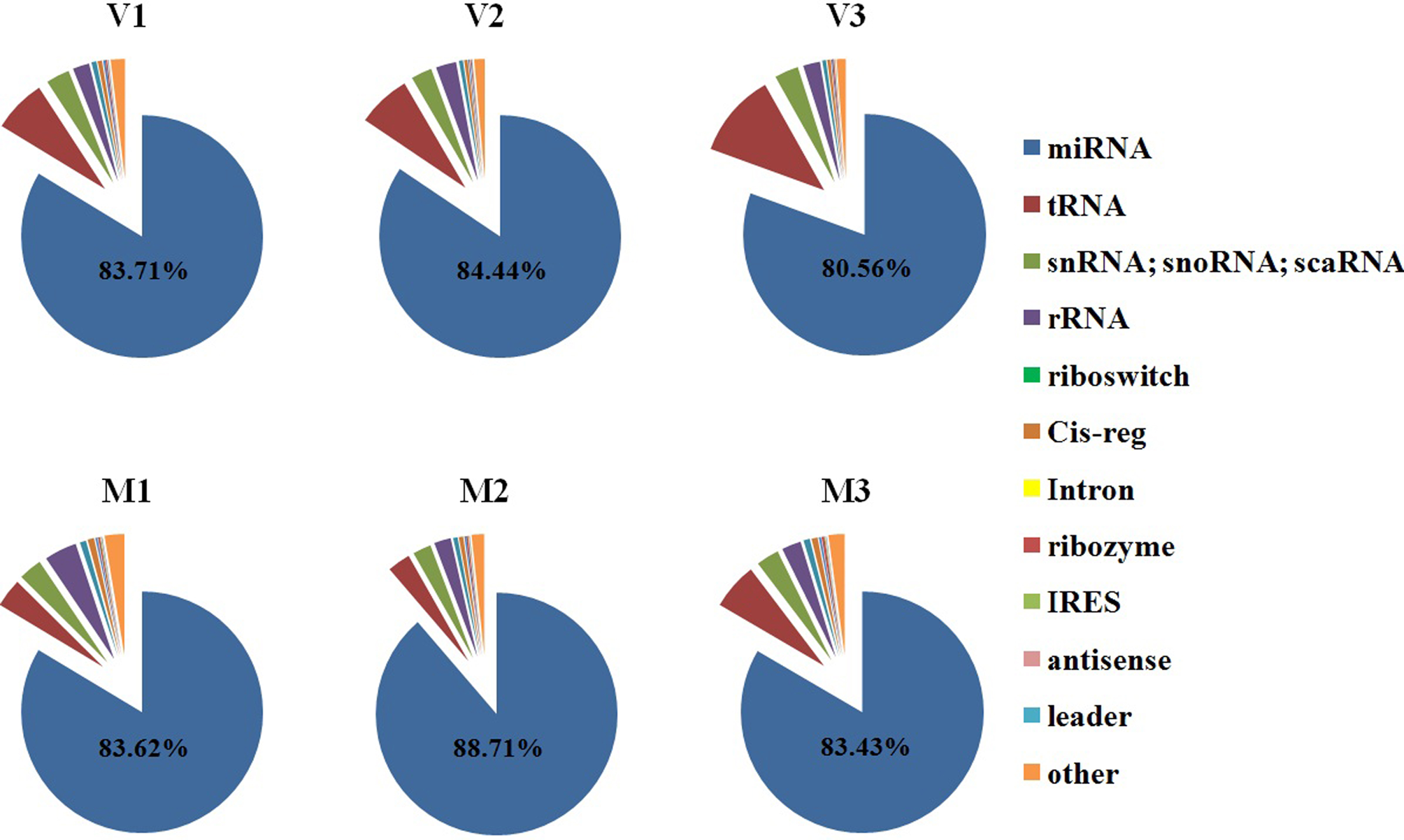

Supplement: Supplementary file 2 — Additional file 2. Pie charts of small RNAs percentages in libraries from H9N2-infected and mock-infected DCs. More than 80% of the annotated small RNAs in all libraries were miRNAs. M: mock-infected DCs; V: H9N2 AIV-infected DCs. [file 13567_2020_856_MOESM2_ESM.tif]
